# Construction and validation of a risk assessment scale for multidrug-resistant bacteria infections in critically ill patients

**DOI:** 10.3389/fmed.2025.1711440

**Published:** 2025-11-14

**Authors:** Jia Zhao, Li Yuan, Yujing Wang, Bing Liu, Xiaoyan Wang, Xuexian Ma, Ping Li

**Affiliations:** 1 School of Nursing, Xinjiang Medical University, Xinjiang, China; 2 The Second Affiliated Hospital of Xinjiang Medical University, Xinjiang, China; 3 Health Care Research Center for Xinjiang Regional Population, Xinjiang, China

**Keywords:** multi-drug resistant bacteria, Delphi method, scale, reliability, validity

## Abstract

**Background:**

To develop a risk assessment scale for infections caused by multidrug-resistant organisms (MDROs) in patients with critical illness and to evaluate its reliability and validity.

**Methods:**

An initial risk assessment scale for MDRO infections in patients with critical illness was developed by using a systematic literature review, the Delphi method, and the analytic hierarchy process. Data from 750 critically ill patients admitted to the intensive care unit (ICU) of a Grade A tertiary hospital in China between January 2019 and June 2025 were analyzed. The scale’s reliability and validity were assessed through exploratory factor analysis (*n* = 450) and confirmatory factor analysis (*n* = 300).

**Results:**

The risk assessment scale for MDRO infection in patients with critical illness comprised five dimensions and 20 items. The Cronbach’s alpha for the total scale was 0.873. The scale-level content validity index was 0.925, with the content validity indices for individual scale items ranging 0.875–1.000. Exploratory factor analysis enabled the extraction of five common factors, which accounted for 67.861% of the cumulative variance. Confirmatory factor analysis yielded *χ*^2^/DF = 1.912, RMSEA = 0.055, CFI = 0.945, TLI = 0.935, and GFI = 0.906. The predictive efficacy of the scale for MDRO infections was validated via ROC curve analysis, yielding an area under the curve (AUC) of 0.788, sensitivity of 0.741, and specificity of 0.707, indicating a robust overall discriminative capability.

**Conclusion:**

The risk assessment scale for MDRO infection in patients with critical illness demonstrated good reliability and validity, enabling the scientific and reliable assessment of patient infection risk.

## 1 Introduction

Infections caused by multidrug-resistant organisms (MDROs) pose a significant challenge for patients with critical illness. MDRO infections have become a major global public health concern owing to their high incidence, high mortality rate, and treatment complexity (1). Based on the research findings, patients with critical illness exhibit reduced resistance to pathogenic bacteria because of impairment of the bodily functions, placing them at a high risk for hospital-acquired infections (2). The acquisition rate of MDRO infections in intensive care units (ICUs) is 41%, and the attributable mortality rate among patients with MDRO infections is considerably higher than that in patients with other types of infections. This high mortality rate not only seriously endangers patients’ lives and health but also imposes a heavy burden on the healthcare system (3).

Despite providing valuable insights into the prevention and control of MDRO infections, the reviewed studies have some limitations, for example, the lack of department-specific and patient-specific focus, as well as the difficulty in translating the results to the frontline clinical setting. This gap between research and practical application ultimately hinders the applicability of the cumulative findings toward guiding clinical interventions. For instance, Liu’s (4) predictive model incorporated only nine risk factors, many of which are difficult to modify through nursing interventions. Furthermore, the model lacks a clear demonstration of the temporal relationship between these factors and the outcome, undermining its predictive validity and clinical applicability. Some research evaluation processes are highly subjective, which makes it difficult to accurately identify high-risk individuals (5, 6). In addition, some MDRO infection risk assessment scales designed for general hospitalized patients do not fully consider the complex and variable characteristics of those with critical illness and hence cannot be effectively applied to this special patient population (7, 8). The National Action Plan for Containment of Microbial Resistance (2022–2025) (9) emphasizes precise prevention and control; however, rapid assessment tools that are quantifiable, provide early warning, and can be used in ICUs are lacking. Therefore, we integrated the Delphi expert consensus with the analytic hierarchy process (AHP) in this study to develop and validate a set of tools for predicting MDRO infection risk stratification, specifically for patients with critical illness. This scale provides a basis for the rapid clinical identification of patients at high risk, scientifically predicts the infection risk levels, and facilitates the timely implementation of the targeted prevention and control measures.

## 2 Materials and methods

### 2.1 Study setting

This study was conducted at a Grade III, Level A tertiary hospital in Urumqi, China, which has a capacity of approximately 800 inpatient beds and is equipped with multiple ICUs, including a general ICU (GICU) and a neurological ICU (NICU).

### 2.2 Participants

This study employed a retrospective design by utilizing clinical data from all patients admitted to the ICUs of the hospital between January 2019 and June 2025. The inclusion criteria were as follows: (1) age ≥18 years; (2) Hospitalization duration ≥48 h, with infections occurring ≥48 h after admission were defined as hospital-acquired infections for pathogens without a defined incubation period (10). Exclusion criteria were as follows: (1) patients with ≥10% missing medical record data; (2) patients who never submitted specimens during hospitalization or showed erroneous test results; (3) pregnant women and women in the puerperium period. The primary outcome event was the development of drug-resistant bacterial infection in ICU patients 48 h after admission.

### 2.3 Pathogen culture and antimicrobial susceptibility testing

Pathogen specimens (e.g., sputum, blood, urine, ascites, drainage fluid, pus, bronchoalveolar lavage fluid, and secretions) were collected and transported as per the standard clinical microbiology procedures (11). Laboratory analysis followed the National Clinical Laboratory Operating Procedures (12). Strain identification was performed using the bioMérieux VITEK-2 compact system. Antimicrobial susceptibility testing was performed by using the minimum inhibitory concentration (MIC) method, interpreted as per the 2021 CLSI guidelines (13) (categorized as susceptible, intermediate, or resistant). MDROs were defined as bacteria resistant to ≥3 antimicrobial classes, including carbapenem-resistant Enterobacteriaceae (CRE), *Klebsiella pneumoniae* (CRKP), *Pseudomonas aeruginosa* (CRPA), methicillin-resistant *Staphylococcus aureus* (MRSA), and carbapenem-resistant *Acinetobacter baumannii* (CRAB). MDRO infection diagnosis adhered to the established technical guidelines (14), requiring clinical assessment and confirmation by the hospital infection control department so as to exclude community-acquired cases, colonization, or contamination.

### 2.4 Methods

**Figure 1:**
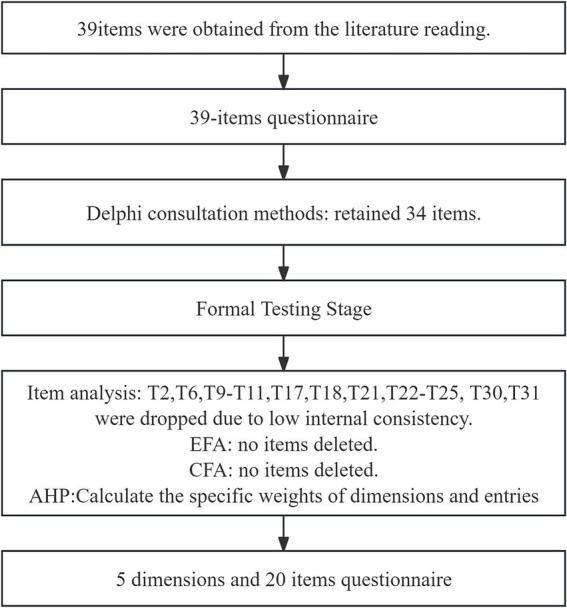
The number of items at each stage of measurement development.

The scale development was informed by a systematic literature review and subsequently refined by using the Delphi method and AHP, as outlined in Figure 1.

#### 2.4.1 Construction of the indicator system

The Chinese search terms “危重患者/重症监护室”, “多重耐药菌”, “感染”, and “影响因素” were searched across databases such as China National Knowledge Infrastructure (CNKI), Wanfang Database, and VIP Database. The English search terms “Critically ill patients/Intensive care unit,” “multi-drug resistant organisms,” “Infection,” and “influencing factors” were searched across English databases such as PubMed, Cochrane Library, and Embase. The search period spanned from the inception of each database to September 2024. After summarizing, concluding, and organizing the existing literature and data, a preliminary version of the MDRO Infection Risk Assessment Form for Critically Ill Patients was drafted. Following the item-by-item discussion and analysis, 5 first-level indicators, which included “patient-related factors,” “disease-related factors,” “biochemical-related factors,” “treatment-related factors,” and “medication-related factors,” as well as 39 s-level indicators, were finally confirmed.

#### 2.4.2 Delphi method

Expert Eligibility Criteria: (1) clinical work experience of at least 10 years; (2) having an intermediate-level professional title or above; (3) possessing a bachelor’s degree or higher; (4) professional expertise covering intensive care, nursing management, and healthcare-associated infection control; (5) voluntary participation with a commitment to complete all rounds of the survey. Questionnaires were distributed and collected by using the following three methods: on-site distribution, WeChat, and email, with a time limit of 2 weeks for each round of consultation. Two rounds of expert consultation were conducted in this study. A total of 20 experts from six distinct regions of China (i.e., Shanxi, Guangxi, Hunan, Beijing, Shanghai, and Xinjiang Uygur Autonomous Region) were invited to participate in the Delphi study. The expert engagement was assessed based on the effective questionnaire response rate. Expert authority was quantified by the authority coefficient (*Cr*), calculated as (*Cs* + *Ca*)/2, where Cs denotes familiarity with the subject and Ca represents the basis of their judgments. The concentration of expert opinions was evaluated by mean importance scores, whereas the degree of consensus was measured by the coefficient of variation (*CV*) and Kendall’s *W*. The screening criteria included the mean score of item importance of ≥3.5 and the coefficient of variation of <0.25.

#### 2.4.3 Data collection

Clinical data were extracted from the Hospital Information System (HIS). The initial scale developed in the earlier phase was administered to ICU patients, with assessments performed at multiple time points: admission, the perioperative period, and during significant clinical or therapeutic changes. Considering that the same patient underwent multiple assessments, subsequent analyses were based on the highest score recorded before the infection.

According to the requirements of factor analysis, the sample size should be at least 5 to 20 times the total number of items in the scale (15). The initial scale in this study comprised 34 secondary items, necessitating a minimum sample size of 170 to 680 cases. To ensure sufficient statistical power for both exploratory and confirmatory analyses, a total of 750 patients were enrolled: 450 for exploratory factor analysis and 300 for confirmatory factor analysis. A comparison of the baseline characteristics between the infected and non-infected groups is presented in Table 1.

**Table 1:** Comparison of baseline characteristics between non-infected and infected groups.

Factor	Category	Non-Infected Group (*n* = 469)	Infected Group (*n* = 281)	*χ*^2^ Value	*p* value
Sex	Male	275 (58.64%)	152 (54.09%)	1.479	0.224
	Female	194 (41.36%)	129 (45.91%)		
Age	< 65	187 (39.87%)	97 (34.52%)	0.061	0.805
	≥ 65	282 (60.13%)	184 (65.48%)		
Employment	Unemployed	186 (39.66%)	114 (40.57%)	2.140	0.144
	Employed	283 (60.34%)	167 (59.43%)		
Marital Status	Single	12 (2.56%)	11 (3.91%)	3.013	0.394
	Married	434 (92.54%)	250 (88.97%)		
	Widowed	18 (3.84%)	16 (5.69%)		
	Divorced	5 (1.07%)	4 (1.42%)		
Number of Hospitalizations	> 1	208 (44.35%)	143 (50.89%)	3.019	0.082
	1	261 (55.65%)	138 (49.11%)		
Route of Admission	Emergency	160 (34.12%)	93 (33.10%)	0.082	0.960
	Outpatient	110 (23.45%)	67 (23.84%)		
	Transfer from other hospital	199 (42.43%)	121 (43.06%)		
Discharge Disposition	Discharge	311 (66.31%)	181 (64.41%)	0.281	0.596
	Death	158 (33.69%)	100 (35.59%)		
APACHE II Score	< 15	269 (57.36%)	141 (50.18%)	3.653	0.056
	≥ 15	200 (42.64%)	140 (49.82%)		

#### 2.4.4 Weight assignment

The AHP was employed to scientifically determine the relative weights of the scale items. For each pairwise comparison matrix, the maximum eigenvalue (*λ* < sub > max</sub>) was calculated. The consistency index (*CI*) was then derived as *CI* = (λ < sub > max</sub > − n) / (n - 1). Finally, the consistency ratio (*CR* = *CI* / *RI*) was computed against Saaty’s random index (*RI*). Only matrices with a *CR* value of <0.10 were retained, ensuring acceptable consistency in the expert judgments (16).

#### 2.4.5 Statistical analysis

Statistical analyses were performed using SPSS (version 26.0) and AMOS (version 23.0). (1) Project analysis: ① Critical Ratio (*CR*) Method: The total scale scores were ranked in descending order. Participants in the top 27% and bottom 27% were assigned to the high-score and low-score groups, respectively. An independent samples t-test was conducted to compare the mean scores of each item between the two groups. Items with a critical ratio (*CR*) value of <3.0 were considered for removal. ② Corrected Item-Total Correlation (*CITC*) Method: Items exhibiting a corrected item-total correlation coefficient of <0.40 were flagged for potential deletion. ③ Homogeneity Test: The internal consistency was assessed by calculating Cronbach’s alpha for the total scale. Items whose deletion increased the overall Cronbach’s alpha coefficient were removed to enhance scale homogeneity. (2) Validity test: ① Structural validity: The structural validity of the scale was examined using both Exploratory Factor Analysis (EFA) and Confirmatory Factor Analysis (CFA). ②Content Validity: Content validity was evaluated by calculating the Content Validity Index at both the item level (I-CVI) and the scale level (S-CVI) (15). (3) Reliability test: Cronbach *α*-coefficient and split-half reliability were selected. (4) AHP: The AHP was conducted using Yaahp V10 and SPSSPRO 1.1.1 software to determine the relative weights of indicators at different levels within the scale framework. (5) Predictive Validity: The predictive validity of the scale for MDRO infection was assessed by using receiver operating characteristic (ROC) curve analysis. The optimal diagnostic cut-off value was determined, and its capability in identifying true-positive cases was evaluated. Continuous data were expressed as the mean and standard deviation ( $\bar{x}$ ± s) and based on the enumeration data as numbers and percentages (%).

## 3 Results

### 3.1 Delphi expert consultation

A total of 20 questionnaires were distributed across two rounds of consultation, all of which were returned and found to be valid, yielding a 100% response rate and a high level of expert engagement. The Kendall’s coefficient of concordance ranged from 0.380 to 0.449 (*p* < 0.05), indicating good consistency among the consultation opinions of experts. The active, authority, and concordance coefficients of the experts in the expert consultation were 0.88, 0.885, and 0.8825, respectively. These figures signify that the experts demonstrated a high level of authority and that the study results were generally reliable. After two rounds of expert correspondence, nine entries were eliminated, two were modified, four were merged into two, six were added, and finally, 34 were retained.

### 3.2 Project analysis results

No items were identified for removal based on the Critical Ratio (CR) method, as all CR values exceeded the threshold of 3.0. The Corrected Item-Total Correlation (CITC) analysis revealed that 16 items (i.e., T2, T6, T9, T10, T11, T16, T17, T18, T20, T21, T22, T23, T24, T25, T30, and T31) exhibited correlations <0.40 criterion. Homogeneity testing indicated that the deletion of 12 specific items (i.e., T2, T6, T9, T10, T11, T17, T21, T23, T24, T25, T30, and T31) would result in a significant increase in the scale’s overall Cronbach’s alpha coefficient. Following some deliberations, items T16 and T20 were retained despite their CITC values being slightly <0.40, as they were very close to this threshold and demonstrated satisfactory performance in both the CR method and homogeneity test. Based on pre-established criteria, a total of 14 items were ultimately eliminated. The final scale retained 20 items. The detailed results are presented in Table 2.

**Table 2:** A summary of the scale item analysis results.

Item	*CR*	*r*	Cronbach α value after item deletion	Screening results
T1 Age	–	0.509	0.833	Retain
T2 Length of hospital stay	–	**0.139**	**0.844**	Eliminate
T3 APACHE II score	–	0.520	0.833	Retain
T4 NRS-2002 score	−27.338	0.475	0.835	Retain
T5 Length of ICU stay	–	0.540	0.832	Retain
T6 GCS score	–	**0.186**	**0.843**	Eliminate
T7 Pneumonia	–	0.484	0.834	Retain
T8 Chronic Obstructive Pulmonary Disease	–	0.465	0.835	Retain
T9 Malignant tumor	−20.155	**0.133**	**0.843**	Eliminate
T10 Hypertension	−18.847	**0.171**	**0.843**	Eliminate
T11 Diabetes mellitus	–	**0.136**	**0.844**	Eliminate
T12 Coronary Artery Disease	–	0.528	0.832	Retain
T13 Cerebrovascular disease	–	0.444	0.835	Retain
T14 Hepatic and renal insufficiency	–	0.487	0.834	Retain
T15 Hypoproteinemia	–	0.443	0.835	Retain
T16 Albumin content	−44.585	**0.380**	0.837	Retain
T17 White blood cell count	–	**0.097**	**0.845**	Eliminate
T18 Interleukin-6	−6.143	**0.209**	0.841	Eliminate
T19 C-reactive protein	−17.002	0.407	0.837	Retain
T20 Procalcitonin	−10.645	**0.373**	0.838	Retain
T21 D-dimer	−4.871	**0.149**	**0.842**	Eliminate
T22 Surgery	−36.813	**0.233**	0.841	Eliminate
T23 Hemodialysis	−24.124	**0.166**	**0.842**	Eliminate
T24 drainage tube	−19.699	**0.100**	**0.844**	Eliminate
T25 Endoscopy	–	**0.161**	**0.843**	Eliminate
T26 Tracheotomy/Tracheal Intubation	–	0.458	0.834	Retain
T27 Duration of mechanical ventilation	–	0.434	0.835	Retain
T28 Duration of central venous catheterization	−28.313	0.426	0.836	Retain
T29 Duration of urinary catheterization	−14.91	0.436	0.836	Retain
T30 Invasive blood pressure monitoring duration	–	**0.134**	**0.844**	Eliminate
T31 Duration of Antimicrobial Use	–	**0.150**	**0.844**	Eliminate
T32 Combination Use of Antimicrobial Agents	−54.547	0.555	0.831	Retain
T33 Duration of Concurrent Antimicrobial Therapy	–	0.470	0.834	Retain
T34 Use of special antimicrobial drugs	–	0.492	0.833	Retain

Some entries exhibited a standard deviation of zero, rendering *t*-tests inapplicable. After verifying the data, no errors were found. A standard deviation of zero itself constitutes a significant analytical finding. It indicates that the item possesses extreme discriminative power but lacks variability. Therefore, it is deemed to pass the critical ratio test. This section is denoted by a hyphen (−). Values in bold are statistical values under consideration for exclusion.

### 3.3 Content validity

I-CVI ranged from 0.875 to 1.000, whereas S-CVI/AVE was 0.925, demonstrating good content validity.

### 3.4 Construct validity

#### 3.4.1 Exploratory factor analysis

The Kaiser–Meyer–Olkin statistic was 0.838 > 0.7, and Bartlett’s sphericity test revealed significant differences (*p* < 0.001), confirming suitability for factor analysis.

Exploratory factor analysis yielded five factors with eigenvalues >1, which collectively accounted for 67.861% of the total variance.

The five factors comprised 20 items, with factor loadings for each item ranging from 0.669 to 0.862. Most of the items included in each factor exhibited a clustered distribution consistent with the theoretical hypotheses, and the dimension division generally agreed with the theoretical assumptions. Based on these findings, the condition and hospitalization status of patients with MDRO infections, and joint discussion with experts, the following five factors were selected: “patient-related factors,” “disease-related factors,” “biochemical-related factors,” “treatment-related factors,” and “medication-related factors.”

#### 3.4.2 Confirmatory factor analysis

χ^2^/DF = 1.912 < 3, RMSEA = 0.055 < 0.08, CFI = 0.945, TLI = 0.935, and GFI = 0.906, all of which exceeded 0.9. The five-factor scale demonstrated good construct validity. All dimensions’ standardized factor loadings exceed 0.6, and each dimension’s CR and AVE meet the standards, as listed in Table 3.

**Table 3:** Results of confirmatory factor analysis.

Dimension	Item	Standardized factor loading	AVE	CR
F1 Patient-related factors	T5	0.643	0.839	0.569
	T4	0.751		
	T3	0.856		
	T1	0.752		
F2 Disease-related factors	T12	0.746	0.869	0.527
	T8	0.629		
	T7	0.687		
	T13	0.781		
	T14	0.809		
	T15	0.686		
F3 Biochemical factors	T20	0.67	0.818	0.602
	T19	0.809		
	T16	0.838		
F4 Treatment-related factors	T28	0.8	0.844	0.575
	T27	0.77		
	T26	0.692		
	T29	0.767		
F5 Drug-related factors	T34	0.767	0.828	0.617
	T33	0.754		
	T32	0.833		

The correlations among the five factors ranged from 0.295 to 0.459. As shown in Table 4, all inter-factor correlations were lower than the square roots of the respective average variance extracted (AVE) values, demonstrating adequate discriminant validity.

**Table 4:** Discriminant validity assessment: square roots of AVEs and factor correlations.

Dimension	F1	F2	F3	F4	F5
F1	0.754				
F2	0.387*	0.726			
F3	0.323*	0.305*	0.776		
F4	0.354*	0.353*	0.295*	0.758	
F5	0.357*	0.459*	0.347*	0.458*	0.785

*p < 0.05; diagonal values represent the square root of AVE.

### 3.5 AHP

This study selected 13 experts from the pool of consultants who completed the Delphi consultation to form the AHP expert panel, based on their expert authority coefficient (*Cr* >0.7) and participation enthusiasm (100% valid response rate for both rounds of Delphi questionnaires). Before formal scoring, all experts received standardized training on the principles of the AHP method and the “1–9 scale method” to ensure consistent understanding of the judgment criteria. Experts conducted pairwise comparisons and scoring for the 20 selected items, and the weights were calculated based on these comparisons. The specific results are shown in Table 5.

**Table 5:** Table of comprehensive weights derived from the AHP method.

Dimension	Relative Weight (W1)	Item	Relative Weight (W2)	Comprehensive Weight (W1*W2)
F1	0.0685	T1	0.1973	0.0135
		T3	0.2407	0.0165
		T4	0.0696	0.0048
		T5	0.4924	0.0337
F2	0.2417	T7	0.1635	0.0395
		T8	0.0916	0.0221
		T12	0.3824	0.0924
		T13	0.0715	0.0173
		T14	0.2517	0.0608
		T15	0.0394	0.0095
F3	0.0662	T16	0.2128	0.0141
		T19	0.0948	0.0063
		T20	0.6925	0.0458
F4	0.2754	T26	0.1117	0.0308
		T27	0.2204	0.0607
		T28	0.1602	0.0441
		T29	0.5078	0.1398
F5	0.3483	T32	0.3432	0.1195
		T33	0.3846	0.1340
		T34	0.2722	0.0948

### 3.6 Predictive validity

**Figure 2:**
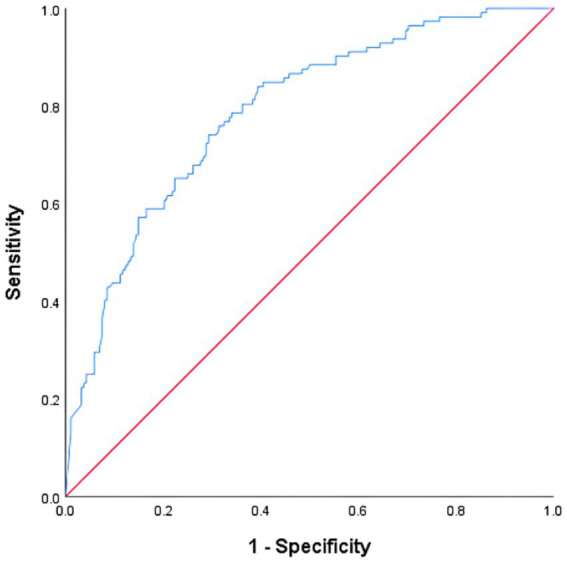
ROC curve of the scale for predicting MDRO infection in patients with critical illness.

To evaluate the predictive efficacy of the constructed scale for MDRO infections in critically ill patients, this study employed ROC curve analysis. The state variable was defined as “whether the patient has an MDRO infection,” while the test variable was the “total scale score.” The area under the curve (AUC) for predicting MDRO infection based on the total scale score was 0.788 (*p* < 0.001). This AUC value was statistically significantly superior to the random classification line (AUC = 0.5), indicating that the scale possesses acceptable discriminatory capability. By calculating the maximum Youden index, the optimal diagnostic cutoff for the total scale score was determined to be 0.843. At this cutoff point, the sensitivity for predicting infection was 0.741, while the specificity was 0.707 (Youden’s index = 0.448). This value indicated that the scale correctly identified 74.1% of MDRO-infected patients while accurately excluding 70.7% of the uninfected patients, demonstrating a good overall discriminatory performance (Figure 2).

### 3.7 Reliability test

The scale demonstrated good reliability. The overall Cronbach’s alpha was 0.873. For the individual factors, Cronbach’s alpha coefficients ranged from 0.779 to 0.865, and split-half reliability coefficients ranged from 0.731 to 0.869.

## 4 Discussion

### 4.1 The MDRO infection risk assessment scale for patients with critical illness demonstrates sound scientific validity

The item pool in this study was constructed through a systematic literature retrieval and quality evaluation process, which is more rigorous than a simple literature review and brainstorming. The research team analyzed, integrated, and rescreened the risk factors from the literature to form the scale item pool (17). The scientific validity and effectiveness of the results from the Delphi method expert consultation were comprehensively evaluated by assessing the experts’ representativeness, participation enthusiasm, authority coefficient, and Kendall’s concordance coefficient (18). Strict selection criteria were applied during the expert consultation phase, and experts from multiple provinces, cities, and regions were invited to participate in the consultation. These experts encompassed multiple fields and disciplines, including infectious diseases, critical care medicine, and the Centers for Disease Control and Prevention. Owing to their extensive theoretical research and practical experience, the experts could provide professional guidance and suggestions. The Kendall’s concordance coefficients for the two rounds of consultation were 0.380 and 0.449 (both *p* < 0.001). This result demonstrated that the experts’ opinions were relatively consistent and concentrated, suggesting a high reliability of the constructed indicator system (19).

AHP was applied in this study to construct hierarchical models and judgment matrices and to calculate the weights of primary and secondary indicators. Using Saaty’s scaling method, the weights were assigned rationally and scientifically to each item and scored. All matrices in the scale passed consistency tests, which enabled quantitative and objective item analysis (20). Of the five primary indicators, drug-related factors carried the highest weight (0.3483), followed by treatment-related factors (0.2754). These results indicated that strictly controlling the duration and type of antimicrobial use is the most critical measure for preventing infections, which agrees with findings from previous studies (21, 22). The evaluation system constructed in this study featured a well-defined hierarchy, a scientifically structured framework, substantial content, and reasonably set weightings, exhibiting a high degree of scientific rigor.

### 4.2 The MDRO infection risk assessment scale for patients with critical illness demonstrates good reliability and validity

Five common factors were identified in this study via multiple rounds of exploratory factor analysis and scree plot assessment. These factors collectively explained 67.861% of the variance. Each item exhibited factor loadings of >0.5 on its assigned common factor without multiple loadings, confirming the robust structural stability of the scale. All fit indices of the confirmatory factor analysis were found to be within the acceptable range, verifying the good construct stability of the scale. The content validity index at the scale level was 0.925, and that for each item ranged from 0.875 to 1.000, indicating that the scale had good content validity (23). The Cronbach’s alpha for the total scale was 0.873, indicating a good internal consistency reliability for both the total scale and all of its dimensions. In conclusion, the development and validation process of the scale was rigorous and standardized, establishing that the scale possessed good reliability.

### 4.3 The MDRO infection risk assessment scale for patients with critical illness exhibits significant clinical utility

In empirical studies, the MDRO infection risk assessment scale for patients with critical illness demonstrated a high clinical utility. This scale featured clear evaluation criteria, a reasonable number of items, straightforward language, and a simple assessment method. Healthcare professionals can quickly grasp and master it, facilitating efficient application in clinical practice. In terms of the dimensions and item settings of the scale, it comprised five dimensions (“patient-related factors,” “disease-related factors,” “biochemical-related factors,” “treatment-related factors,” and “medication-related factors”) with 20 items, comprehensively and systematically covering various factors likely involved in the occurrence of MDRO infections in patients with critical illness. Unlike certain existing scales, this scale not only focuses on patients’ underlying diseases and physical function indicators but also incorporates key information such as the duration of specific antimicrobial use and invasive blood pressure monitoring. This scale systematically and practically considers the specific risk factors for the special population of patients with critical illness, aligning with clinical realities (24, 25). The use of special antimicrobials is an independent risk factor for MDRO infections in patients with critical illness. To rapidly control infections and prevent disease progression, special antimicrobials are commonly used in these patients, and the combination use of such antimicrobials may even be adopted (26). The risk assessment scale for MDRO infections in patients with critical illness developed in this study exhibited high specificity as it fully considered the characteristics of patients with critical illness and infection-related factors. Furthermore, ROC curve analysis confirmed the scale’s predictive utility for MDRO infection. At the optimal cut-off value of 0.843, the scale demonstrated a balanced sensitivity of 0.741 and specificity of 0.707, indicating that, in clinical practice, the tool can correctly identify 74.1% of infected patients while accurately ruling out 70.7% of non-infected patients. This balance is crucial, as the high sensitivity enables effective screening and early warning, whereas the specificity of >70% helps control false positives, preventing unnecessary interventions and optimizing resource allocation.

### 4.4 Limitations

This study is a single-center retrospective study, and the sample source has certain limitations. Therefore, multicenter prospective studies must be conducted in the future to ascertain its validity. The next step is to develop this scale into an electronic risk assessment system, embed it within the hospital information system, and integrate the clinical decision support system function. These measures will enable the development of early warning systems for real-time and dynamic risk, providing an intelligent tool for precise prevention and control.

## 5 Conclusion

This study focused on the construction and validation of a risk assessment scale for MDRO infections in patients with critical illness and analyzed its clinical application value. The results showed that the finally developed scale comprised five dimensions, namely “patient-related factors,” “disease-related factors,” “biochemical-related factors,” “treatment-related factors,” and “medication-related factors,” with a total of 20 items. The total scale demonstrated good reliability and validity, with a Cronbach’s *α* coefficient of 0.873. Furthermore, the scale exhibited strong clinical utility, as evidenced by an area under the ROC curve of 0.788 and an optimal cutoff value of 0.843 points, enabling effective differentiation between infected and noninfected populations. Of the various dimensions, “medication-related factors” was the key influencing dimension.

Therefore, healthcare professionals can use this scale for the routine assessment of MDRO infection risk in patients with critical illness, with particular attention to those on prolonged antimicrobial therapy and those with complex underlying diseases. In clinical interventions, targeted prevention and control measures can be implemented based on the risk stratification results of the scale. For instance, the monitoring of antimicrobial agents for patients at high risk can be strengthened, and antimicrobial management can be optimized for those at low-to-moderate risk. Such strategies can facilitate the precise identification of high-risk populations for MDRO infections, reduce infection rates among patients with critical illness, alleviate the burden on healthcare systems, and provide practical support for implementing the National Action Plan for Combating Microbial Resistance (2022–2025).

## Data Availability

The data analyzed in this study is subject to the following licenses/restrictions: with the consent of the corresponding author and not for commercial use. Requests to access these datasets should be directed to PL, 894088343@qq.com.
