# Supplementary material for: Construction and validation of a risk assessment scale for multidrug-resistant bacteria infections in critically ill patients
Source: Front Med (Lausanne). 2025 Nov 14;12:1711440. doi: 10.3389/fmed.2025.1711440 (PMC12660218; doi:10.3389/fmed.2025.1711440)
Supplement: Supplementary file 1 [file Supplementary_file_1.docx]

**Analytic Hierarchy Process**

1.Introduction to the Analytic Hierarchy Process and Its Procedure

The Analytic Hierarchy Process (AHP), developed by American operations researcher T. L. Saaty in the 1970s, is a multi-criteria decision-making (MCDM) tool that effectively integrates qualitative and quantitative analyses. Its core principle involves decomposing a complex problem into a hierarchical structure (e.g., objectives, criteria, and alternatives), determining the relative importance of elements through pairwise comparisons, and then employing mathematical methods to calculate their weights to inform decision-making. This study applied AHP to determine the weights of the indicators. The specific procedure is illustrated in the flowchart and described in the following steps:

Fig S1.


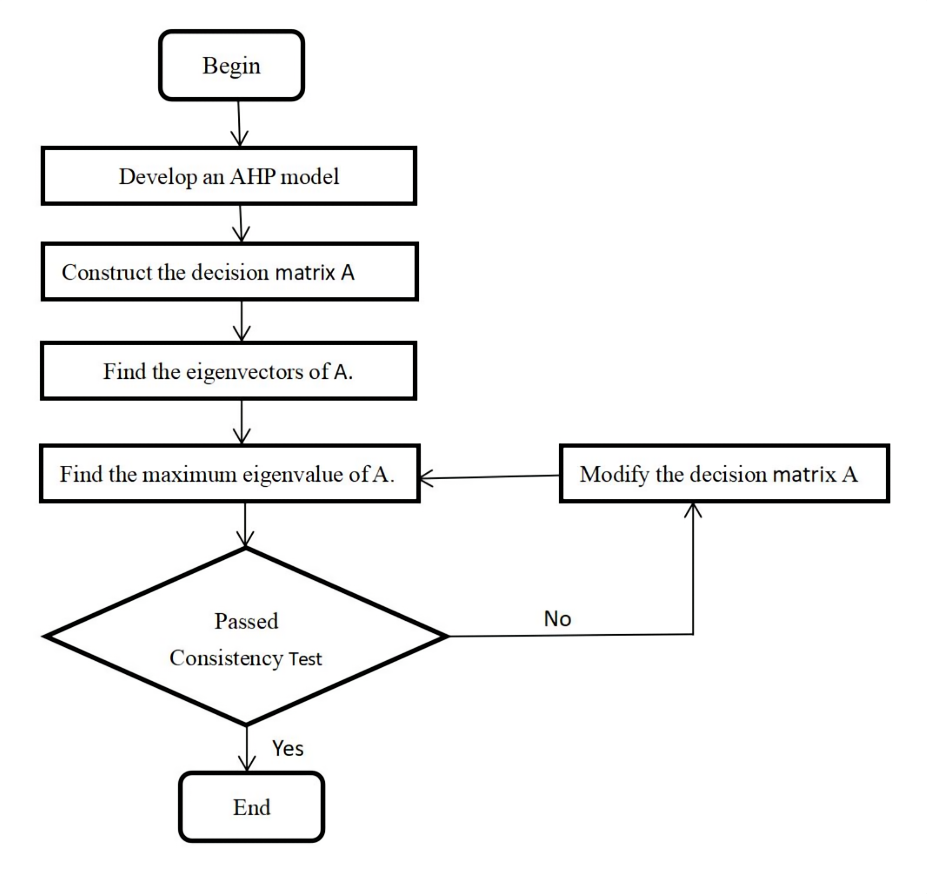


Fig S1.Analytic Hierarchy Process Calculation Flowchart

1.1 Establishing a Hierarchical Analysis Structure Model

The application of the Analytic Hierarchy Process (AHP) begins with structuring the problem into a hierarchy based on a predefined evaluation framework. The overall objective is decomposed into a multi-level model by breaking it down into its constituent factors (e.g., first-level and second-level indicators), organized according to their interrelationships. This results in a top-down hierarchy comprising the goal layer, criterion layers, and sub-criteria layers, as detailed in Table S1.

Table S1

| Primary indicator | Secondary Indicators |
| --- | --- |
| F1 Patient-related factors | T1 Age |
|  | T3 APACHE II score |
|  | T4 NRS-2002 score |
|  | T5 Length of ICU stay |
| F2 Disease-related factors | T7 Pneumonia |
|  | T8 Chronic Obstructive Pulmonary Disease |
|  | T12 Coronary Artery Disease |
|  | T13 Cerebrovascular disease |
|  | T14 Hepatic and renal insufficiency |
|  | T15 Hypoproteinemia |
| F3 Biochemical factors | T16 Albumin content |
|  | T19 C-reactive protein |
|  | T20 Procalcitonin |
| F4 Treatment-related factors | T26 Tracheotomy/Tracheal Intubation |
|  | T27 Duration of mechanical ventilation |
|  | T28 Duration of central venous catheterization |
|  | T29 Duration of urinary catheterization |
| F5 Drug-related factors | T32 Combination Use of Antimicrobial Agents |
|  | T33 Duration of Concurrent Antimicrobial Therapy |
|  | T34 Use of special antimicrobial drugs |

Table S1 Hierarchical Framework Table

1.2 Constructing the Judgment Matrix

Directly quantifying the relative importance of factors poses a significant challenge. To address this, the Analytic Hierarchy Process (AHP) employs pairwise comparisons to construct a judgment matrix. The 9-point scale method is used for these comparisons, which employs 17 discrete values—the integers 1 through 9 and their reciprocals—to define the value of each element a_ij_ in the matrix. The specifics of this scale are detailed in Table S2

Table S2

| Serial Number | Scale Meaning | Ratio |
| --- | --- | --- |
| 1 | When comparing the former element i with the latter element j, i and j are equally important. | a_ij_=1 |
| 2 | When comparing the former element i with the latter element j, i is slightly more important than j. | a_ij_=3 |
| 3 | When comparing the former element i with the latter element j, i is significantly more important than j. | a_ij_=5 |
| 4 | When comparing the preceding element i with the subsequent element j, i is significantly more important than j. | a_ij_=7 |
| 5 | When comparing the preceding element i with the subsequent element j, i is absolutely more important than j. | a_ij_=9 |
| 6 | Indicates that the importance of element i relative to element j falls between the above judgments. | a_ij_=2,4,6,8 |
| 7 | If the relative importance scale of element i to element j is denoted as a_ij_, then the relative importance scale of element j to i is a_ji_=1/ a_ij_ | Countdown |

Table S2 Scaling Method Table

Let the n factors associated with the upper-level factor z be denoted as x1，x2，…..xn ,where i, j = 1, 2, …, n. Let a represent the ratio of the influence of x to x with respect to z. This yields a judgment matrix for pairwise comparisons of these n factors relative to z.Thus, we obtain the judgment matrix $A_{n\times n}$ for pairwise comparisons of these n factors with respect to z.

$A_{n\times n}=\left[ \begin{matrix} a_{11} & a_{12} & a_{1..} & a_{1n} \\ a_{21} & a_{22} & a_{2..} & a_{2n} \\ a_{..} & a_{..} & a_{..} & a_{..} \\ a_{n1} & a_{n2} & a_{n..} & a_{\mathrm{nn}} \end{matrix} \right]$ （1）

1.3 Combining expert matrices using the geometric mean method ensures consistency verification. The scoring matrices formed by m experts (m=1,2,..k) are multiplied element-wise, then raised to the mth power to obtain a unique ensemble matrix $\overline{A}$, as shown in the following formula:

$\overline{A}=(\prod_{k=1}^{m} a_{ij}^{k})^{\frac{1}{m}}$ （2）

1.4 Calculating Relative Weights in Judgment Matrices

Relative weight refers to the importance weight calculated from a judgment matrix, representing the relative significance of a factor at a certain level compared to a factor at the preceding level.

Based on matrix theory, the weights are calculated using the geometric mean method (root method) for the integrated unique matrix, as follows:

$W_{i}=\frac{(\prod_{j=1}^{n} a_{ij})^{\frac{1}{n}}}{\sum_{i=1}^{n} (\prod_{j=1}^{n} a_{ij})^{\frac{1}{n}}} , i=1,2,3..,n$ （3）

Calculation Steps:

① Multiply the elements of vector A row-wise to obtain a new vector;

② Raise each component of the new vector to the nth power;

③ Normalize the resulting vector to obtain the weight vector.

1.5 Consistency Ratio Test for Judgment Matrices

When constructing judgment matrices, due to the complexity of phenomena and the diversity of human subjective perceptions, it is impossible to provide precise ratios between two factors; only estimated judgments can be made. Consequently, the ratios presented in the judgment matrix deviate from actual values, making it impossible to guarantee complete consistency of the matrix. Academic circles generally use *CR* as the standard for assessing judgment matrix consistency. *CR* is the ratio of the consistency index *CI* to the random consistency index *RI*. If *CR* < 0.1, the matrix meets requirements and requires no modification; otherwise, experts should revise the judgment matrix until the calculated *CR* < 0.1.

1.5.1 The formula for calculating the consistency index CI is shown in Equation (4).

$CI=\frac{\lambda_{max}-n}{(n-1)}$ (4)

1.5.2 The CR consistency test calculation formula is shown in Equation (5).

$\text{CR}\text{=}\frac{\text{CI}}{\text{RI}}\text{=}\frac{\text{λ}_{\text{max}}\text{−}\text{n}}{\text{(}\text{n}\text{−1)}\text{RI}}\text{<0.1}$ (5)

1.5.3 λ_max_ represents the maximum eigenvalue of the decision matrix, calculated as shown in Equation (6). λ_max_ is the maximum eigenvalue of the matrix, where $\overline{\text{A}}$ denotes the decision ensemble matrix, W denotes the weight vector, and [$\overline{\text{A}}$W]_i_ denotes the i-th component of matrix [$\overline{\text{A}}$W].

$\lambda_{max}=\sum_{i=1}^{n} \frac{{[\overline{A}W]}_{i}}{{nW}_{i}}$ （6）

1.5.4 In the formula, *RI* is the average consistency index. The *RI* value is related to the order of the matrix, with specific values shown in Table S3.

Table S3

| Matrix order | 1 | 2 | 3 | 4 | 5 | 6 | 7 | 8 | 9 | 10 | 11 | 12 |
| --- | --- | --- | --- | --- | --- | --- | --- | --- | --- | --- | --- | --- |
| *RI* | 0 | 0 | 0.52 | 0.89 | 1.12 | 1.26 | 1.36 | 1.41 | 1.46 | 1.49 | 1.52 | 1.54 |

Table S3 Average Consistency Metrics Table

**2.Data computation**

2.1 Calculation of Primary Indicator Weights

Weights were calculated for primary indicator Patient-Related Factors- Drug-Related Factors. After ensuring the 13 expert judgment matrices met consistency requirements (*CR* < 0.1), the matrices were aggregated using Formula 2. The aggregated matrix is presented in Table S4:

Table S4

| Target Layer | F1 | F2 | F3 | F4 | F5 |
| --- | --- | --- | --- | --- | --- |
| F1 | 1.0000 | 0.2470 | 1.1171 | 0.2290 | 0.2265 |
| F2 | 4.0481 | 1.0000 | 3.7166 | 0.8006 | 0.6515 |
| F3 | 0.8952 | 0.2691 | 1.0000 | 0.2272 | 0.2211 |
| F4 | 4.3668 | 1.2490 | 4.4023 | 1.0000 | 0.6284 |
| F5 | 4.4148 | 1.5349 | 4.5234 | 1.5914 | 1.0000 |

Table S4 Patient-Related Factors - Drug-Related Factors Decision Matrix

Perform weight and consistency calculations on the judgment integration matrix for F1-F5 based on formulas (3), (4), (5), and (6).

FI-F5 Weight calculation results are shown in Table S5.

Table S5

| Target Layer | Weight | *λ_max_* | *CI* | *CR* |
| --- | --- | --- | --- | --- |
| F1 | 0.0685 | 5.0287 | 0.0072 | 0.0064 |
| F2 | 0.2417 |  |  |  |
| F3 | 0.0662 |  |  |  |
| F4 | 0.2754 |  |  |  |
| F5 | 0.3483 |  |  |  |

Table S5 F1 - F5 Weight Calculation Results

2.2 Calculation of Secondary Indicator Weights

Following the same calculation steps as for primary indicators, the judgment integration matrix and weight calculation results for secondary indicators are as follows:

2.2.1 F1 Patient-Related Factors

Weight calculations were performed for the secondary indicators. After ensuring that the judgment matrices from all 13 experts met the consistency requirement (*CR* < 0.1), the 13 individual matrices were consolidated into a single composite matrix. The consolidated matrix is presented in Table S6.

Table S6

| F1 | T1 | T3 | T4 | T5 |
| --- | --- | --- | --- | --- |
| T1 | 1.0000 | 0.8150 | 3.8033 | 0.3002 |
| T3 | 1.2270 | 1.0000 | 3.5689 | 0.4706 |
| T4 | 0.2629 | 0.2802 | 1.0000 | 0.1960 |
| T5 | 3.3312 | 2.1249 | 5.1021 | 1.0000 |

Table S6 Determination Matrix

According to the formula, the weight and consistency of the judgment matrix are calculated, and the weight calculation results are shown in Table S7.

Table S7

| F1 | Weight | *λ_max_* | *CI* | *CR* |
| --- | --- | --- | --- | --- |
| T1 | 0.1973 | 4.0703 | 0.0234 | 0.0263 |
| T3 | 0.2407 |  |  |  |
| T4 | 0.0696 |  |  |  |
| T5 | 0.4924 |  |  |  |

Table S7 Weight calculation results

2.2.2 F2 Disease-Related Factors

Weights were calculated for the secondary indicators. After ensuring that the judgment matrices from all 13 experts met the consistency requirement (*CR* < 0.1), the 13 individual matrices were consolidated into a single matrix. The consolidated matrix is presented in Table S8.

Table S8

| F2 | T7 | T8 | T12 | T13 | T14 | T15 |
| --- | --- | --- | --- | --- | --- | --- |
| T7 | 1.0000 | 1.2290 | 0.4438 | 2.8761 | 0.6276 | 4.7802 |
| T8 | 0.8137 | 1.0000 | 0.1816 | 1.6079 | 0.2655 | 2.3073 |
| T12 | 2.2532 | 5.5072 | 1.0000 | 4.8977 | 2.0634 | 6.1443 |
| T13 | 0.3477 | 0.6219 | 0.2042 | 1.0000 | 0.2661 | 2.8044 |
| T14 | 1.5934 | 3.7667 | 0.4846 | 3.7584 | 1.0000 | 5.7261 |
| T15 | 0.2092 | 0.4334 | 0.1628 | 0.3566 | 0.1746 | 1.0000 |

Table S8 Determination Matrix

According to the formula, the weight and consistency of the judgment matrix are calculated, and the weight calculation results are shown in Table S9.

Table S9

| F2 | Weight | *λ_max_* | *CI* | *CR* |
| --- | --- | --- | --- | --- |
| T7 | 0.1635 | 6.1616 | 0.0323 | 0.0256 |
| T8 | 0.0916 |  |  |  |
| T12 | 0.3824 |  |  |  |
| T13 | 0.0715 |  |  |  |
| T14 | 0.2517 |  |  |  |
| T15 | 0.0394 |  |  |  |

Table S9 Weight calculation results

2.2.3 F3 Biochemical-Related Factors

Weights were calculated for the secondary indicators. After ensuring that the judgment matrices from all 13 experts met the consistency requirement (*CR* < 0.1), the 13 individual matrices were consolidated into a single matrix. The consolidated matrix is presented in Table S10.

Table S10

| F3 | T16 | T19 | T20 |
| --- | --- | --- | --- |
| T16 | 1.0000 | 2.7268 | 0.2531 |
| T19 | 0.3667 | 1.0000 | 0.1661 |
| T20 | 3.9509 | 6.0192 | 1.0000 |

Table S10 Determination Matrix

According to the formula, the weight and consistency of the judgment matrix are calculated, and the weight calculation results are shown in Table S11.

Table S11

| F3 | Weight | *λ_max_* | *CI* | *CR* |
| --- | --- | --- | --- | --- |
| T16 | 0.2128 | 3.0378 | 0.0189 | 0.0363 |
| T19 | 0.0948 |  |  |  |
| T20 | 0.6925 |  |  |  |

Table S11 Weight calculation results

2.2.4 F4 Treatment-Related Factors

Weights were calculated for the secondary indicators. After ensuring that the judgment matrices from all 13 experts met the consistency requirement (*CR* < 0.1), the 13 individual matrices were consolidated into a single matrix. The consolidated matrix is presented in Table S12.

Table S12

| F4 | T26 | T27 | T28 | T29 |
| --- | --- | --- | --- | --- |
| T26 | 1.0000 | 0.3634 | 0.9609 | 0.2225 |
| T27 | 2.7516 | 1.0000 | 1.3961 | 0.3069 |
| T28 | 1.0407 | 0.7163 | 1.0000 | 0.4409 |
| T29 | 4.4939 | 3.2583 | 2.2680 | 1.0000 |

Table S12 Determination Matrix

According to the formula, the weight and consistency of the judgment matrix are calculated, and the weight calculation results are shown in Table S13.

Table S13

| F4 | Weight | *λ_max_* | *CI* | *CR* |
| --- | --- | --- | --- | --- |
| T26 | 0.1117 | 4.1125 | 0.0375 | 0.0421 |
| T27 | 0.2204 |  |  |  |
| T28 | 0.1602 |  |  |  |
| T29 | 0.5078 |  |  |  |

Table S13 Weight calculation results

2.2.5 F5 Drug-Related Factors

Weights were calculated for the secondary indicators. After ensuring that the judgment matrices from all 13 experts met the consistency requirement (*CR* < 0.1), the 13 individual matrices were consolidated into a single matrix. The consolidated matrix is presented in Table S14.

Table S14

| F5 | T32 | T33 | T34 |
| --- | --- | --- | --- |
| T32 | 1.0000 | 0.8770 | 1.2833 |
| T33 | 1.1403 | 1.0000 | 1.3887 |
| T34 | 0.7792 | 0.7201 | 1.0000 |

Table S14 Determination Matrix

According to the formula, the weight and consistency of the judgment matrix are calculated, and the weight calculation results are shown in Table S15.

Table S15

| F5 | Weight | *λ_max_* | *CI* | *CR* |
| --- | --- | --- | --- | --- |
| T32 | 0.3432 | 3.0003 | 0.0002 | 0.0003 |
| T33 | 0.3846 |  |  |  |
| T34 | 0.2722 |  |  |  |

Table S15 Weight calculation results

3.Weight Aggregation

Based on the assessment of each indicator, the calculation results for the integration matrix and weights are summarized in Table 16, which aggregates the weights between indicators at different levels.

Table S16.

| Dimension | Relative Weight (*W1*) | Item | Relative Weight (*W2*) | Comprehensive Weight(*W1*W2*) |
| --- | --- | --- | --- | --- |
| F1 | 0.0685 | T1 | 0.1973 | 0.0135 |
|  |  | T3 | 0.2407 | 0.0165 |
|  |  | T4 | 0.0696 | 0.0048 |
|  |  | T5 | 0.4924 | 0.0337 |
| F2 | 0.2417 | T7 | 0.1635 | 0.0395 |
|  |  | T8 | 0.0916 | 0.0221 |
|  |  | T12 | 0.3824 | 0.0924 |
|  |  | T13 | 0.0715 | 0.0173 |
|  |  | T14 | 0.2517 | 0.0608 |
|  |  | T15 | 0.0394 | 0.0095 |
| F3 | 0.0662 | T16 | 0.2128 | 0.0141 |
|  |  | T19 | 0.0948 | 0.0063 |
|  |  | T20 | 0.6925 | 0.0458 |
| F4 | 0.2754 | T26 | 0.1117 | 0.0308 |
|  |  | T27 | 0.2204 | 0.0607 |
|  |  | T28 | 0.1602 | 0.0441 |
|  |  | T29 | 0.5078 | 0.1398 |
| F5 | 0.3483 | T32 | 0.3432 | 0.1195 |
|  |  | T33 | 0.3846 | 0.1340 |
|  |  | T34 | 0.2722 | 0.0948 |

Table S16 Table of Comprehensive Weights Derived from the AHP Method.

**Definition of Key Concepts**

TableS17

| Name | Definition |
| --- | --- |
| Special Antimicrobial Agents | Special antimicrobial agents^[1]^ refer to antimicrobial drugs that meet any of the following criteria: (1) Strictly restricted use to prevent rapid bacterial resistance development. (2) Significant or severe adverse reactions, making indiscriminate use inadvisable. (3) The antimicrobial agent is prohibitively expensive. (4) Limited clinical data exist regarding the safety and efficacy of the antimicrobial agent. Commonly used special antimicrobial agents in clinical practice include imipenem/cilastatin sodium, meropenem, vancomycin, polymyxin B, and linezolid. |
| Combination Therapy Standards | Indications for Combination Antimicrobial Therapy ^[2]^: Combination therapy is indicated only in the following situations, as infections effectively treatable with a single agent do not require combination therapy.  (1) Severe infections where the causative organism remains unidentified, including severe infections in immunocompromised patients.  (2) Mixed aerobic and anaerobic infections, or infections caused by two or more pathogens, where a single antimicrobial agent is ineffective.  (3) Severe infections such as infective endocarditis or sepsis that cannot be effectively controlled by a single antimicrobial agent.  (4) Infections requiring prolonged treatment where pathogens are prone to developing resistance to certain antimicrobials, such as tuberculosis and deep fungal infections.  (5) When combining drugs with synergistic antibacterial effects, reduce the dosage of highly toxic agents. For example, when treating cryptococcal meningitis with amphotericin B and flucytosine, the amphotericin B dose may be appropriately reduced to minimize toxic reactions. Combination therapy should prioritize drugs with synergistic or additive antibacterial effects, such as penicillins, cephalosporins, and other β-lactams combined with aminoglycosides, or amphotericin B with flucytosine. Typically, two drugs are combined; tri- or multi-drug combinations are reserved for specific indications, such as tuberculosis treatment. In addition, adverse drug reactions may increase following combination therapy. |
| Antibiotic treatment regimen | Duration of Antimicrobial Therapy^[2]^: The duration of antimicrobial therapy varies with the infection. Generally, treatment should continue for 72–96 h after the return to normal body temperature and resolution of symptoms. Special circumstances require appropriate management. However, conditions such as sepsis, infective endocarditis, purulent meningitis, typhoid fever, brucellosis, osteomyelitis, streptococcal pharyngitis and tonsillitis, deep fungal infections, and tuberculosis require extended treatment courses to achieve complete cure and prevent recurrence. |
| **References:**  [1]Ministry of Health of the People's Republic of China. Administrative Measures for the Clinical Application of Antibacterial Drugs (2012). Available online at: <https://www.gov.cn/gongbao/content/2012/content_2201890.htm> (accessed online at October 20, 2025).  [2]The Revision Working Group of the “Guiding Principles for the Clinical Application of Antibacterial Drugs”. Guidelines for the Clinical Application of Antibacterial Drugs (2015 Edition). Beijing: People's Medical Publishing House (2015). | |

TableS17 Definition Supplement Table
